# Emergence of Letermovir-resistant HCMV UL56 mutant during rescue treatment in a liver transplant recipient with ganciclovir-resistant infection HCMV: a case report

**DOI:** 10.1186/s12879-021-06694-4

**Published:** 2021-09-23

**Authors:** Stefania Paolucci, Giulia Campanini, Irene Cassaniti, Alessandra Tebaldi, Federica Novazzi, Alice Fratini, Antonella Meini, Federica Girelli, Laura Palumbo, Alessandro Plebani, Fausto Baldanti

**Affiliations:** 1 Molecular Virology Unit, Microbiology and Virology Department, Fondazione IRCCS Policlinico San Matteo, Pavia, Italy; 2 Unit of Infectious Diseases, ASST Papa Giovanni XXIII, 24129 Bergamo, Italy; 3 Pediatrics Clinic, Department of Clinical and Experimental Sciences, University of Brescia, ASST Spedali Civili of Brescia, Brescia, Italy; 4 Department of Clinical, Surgical, Diagnostic and Pediatric Sciences, University of Pavia, Pavia, Italy

**Keywords:** Case report, HCMV, Letermovir

## Abstract

**Background:**

Human Cytomegalovirus (HCMV) still represents a crucial concern in solid organ transplant recipients (SOTRs) and the use of antiviral therapy are limited by side effects and the selection of viral mutations conferring antiviral drug resistance.

**Case presentation:**

Here we reported the case of an HCMV seronegative patient with common variable immunodeficiency (CVID), multiple hepatic adenomatosis, hepatopulmonary syndrome and portal hypertension who received a liver transplant from an HCMV seropositive donor. The patient was treated with Valganciclovir (vGCV) and then IV Ganciclovir (GCV) at 5 week post-transplant for uncontrolled HCMV DNAemia. However, since mutation A594V in UL97 gene conferring resistance to ganciclovir was reported, GCV therapy was interrupted. Due to the high toxicity of Foscarnet (FOS) and Cidofovir (CDV), Letermovir (LMV) monotherapy at the dosage of 480 mg per day was administered, with a gradual viral load reduction. However, a relapse of HCMV DNAemia revealed the presence of mutation C325Y in HCMV UL56 gene conferring resistance to LMV.

**Conclusions:**

In conclusion, even if LMV is an effective and favorable safety molecule it might have a lower genetic barrier to resistance. A warning on the use of LMV monotherapy as rescue treatments for HCMV GCV-resistant infections in transplant recipients is warranted.

## Background

Antiviral drugs for treatment of systemic human cytomegalovirus (HCMV) infection in immunocompromised patients include viral DNA synthesis inhibitors Ganciclovir (GCV)/Valganciclovir (vGCV), Foscarnet (FOS) and Cidofovir (CDV). However, these drugs are limited by significant side effects and the selection of viral mutations conferring antiviral drug resistance [1]. While GCV-resistant HCMV infections represent a crucial issue in transplant setting, being associated with higher risk of recurrent HCMV disease and high mortality rate [2, 3], FOS and CDV are used as rescue drugs for the treatment of GCV-resistant HCMV infections, because of their renal toxicity. Recently, new drugs with innovative mechanisms of action have been introduced in clinical practice, including Letermovir (LMV) which block the HCMV viral terminase complex and thus inhibits the cleavage/packaging of viral genomes [4, 5]. To date, due to very favorable safety profile, LMV has been approved for prophylaxis in hematopoietic stem cell transplant recipients (HSCTRs) [6]. On the other hand, some studies suggest that LMV might have a lower genetic barrier to resistance [7, 8]. Here, we report the rapid emergence of a LMV-resistant HCMV mutant in a liver transplant recipient undergoing LMV rescue treatment because of GCV-resistant HCMV infection, severe myelosuppression and kidney function impairment. This report is a warning on the use of LMV in monotherapy.

## Case presentation

**Fig. 1:**
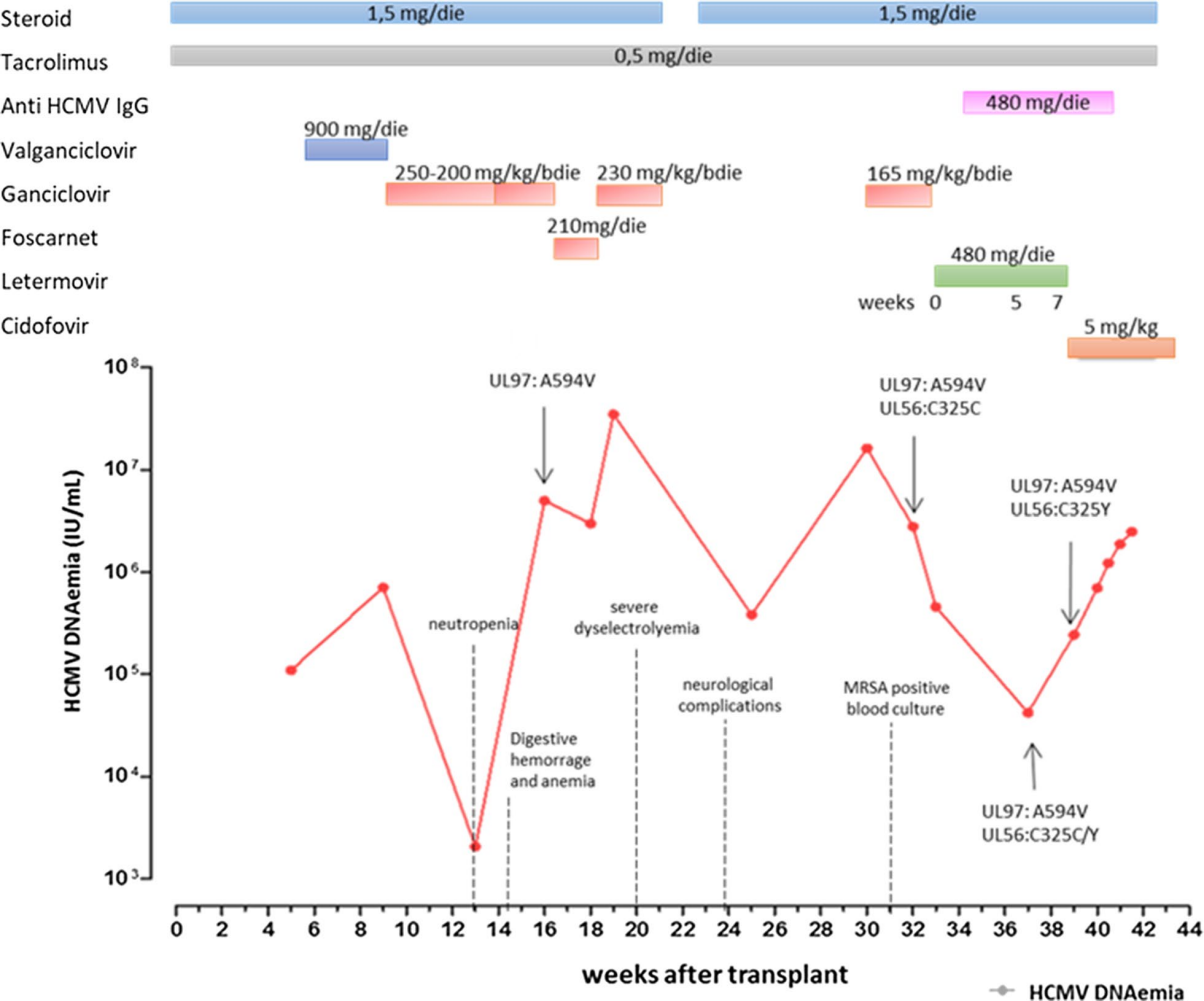
HCMV DNAemia levels in IU/mL detected in whole blood (red line) are shown. Moreover, timeline of medication, including immunosuppressive regimens (tacrolimus and steroid) as well as antiviral therapies (vGCV, GCV, FOS, CDV, LMV and anti-HCMV IgG) is given in the graph. Occurrence of drug resistance (GCV and LMV) are also shown

A 23-year-old woman with common variable immunodeficiency (CVID) was affected by multiple hepatic adenomatosis complicated by hepatopulmonary syndrome and portal hypertension. Renal dysfunction, fungal skin infections, sinusitis, otomastoiditis, and hypothyroidism were also documented. Immunoglobulins substitution therapy and replacement therapy for hypothyroidism were also administered. In October 24 2018, she received an orthotropic liver transplant. At the transplantation baseline, the patient was HCMV seronegative while her donor was HCMV-seropositive. Immunosuppressive therapy included steroids (5 mg/day) and tacrolimus (0.5 mg/day) (Fig. 1). HCMV DNAemia of 111,000 UI/mL was reported 5 weeks post-transplant. VGCV treatment (900 mg twice daily) was introduced. After 11 weeks of vGCV treatment viral load continued to increase up to 713,567 UI/mL, thus intravenous (IV) GCV treatment at 250 mg twice daily was introduced two weeks later, IV GCV dosage was reduced at 200 mg twice daily to minimize myelotoxicity. However, HCMV DNAemia after 13 weeks of treatment was still positive (2076 IU/mL). Colonoscopy and endoscopy performed in order to exclude HCMV-related enteritis did not show relevant finding. At that time, therapy was interrupted due to the worsening of neutropenia.

At week 14, the patient was hospitalized for digestive hemorrhage and anemia. Then, she was transferred at Stroke Unit for limb hyposthenia due to concomitant cerebral ischemic event.

At week 16, HCMV DNAemia increased up to 3,000,000 UI/mL and mutation A594V in UL97 gene conferring resistance to GCV and vGCV was documented [9]. UL54 gene analysis excluded FOS resistance [10], thus FOS treatment (90 mg/kg three times daily) was initiated. Unfortunately, due to the onset of severe dyselectrolyemia, FOS was discontinued after 10 days of treatment and a rapid increased of HCMV DNAemia (35,000,000 IU/mL) was observed. GCV was initiated at the dosage of 230 mg twice daily but it was suspended after two weeks due to the very limited effect on HCMV DNAemia and worsening of anemia and thrombocytopenia.

At week 20, since the patient was highly immunosuppressed, tacrolimus was reduced and steroid was suspended. However, steroid had to be reintroduced after one week due to the onset of neurological complications. At that time, HCMV specific T-cell response determined by ELISpot assay [11] was undetectable, since the patient showed 91 CD4 T cells/µl, and 71 CD8 T cells/µl.

At week 21 the patient was hospitalized due to a respiratory distress, caused by HCMV lung infection and a methicillin-resistant Staphylococcus aureus (MRSA) septicemia.

GCV therapy was reintroduced in combination with HCMV-specific immunoglobulin therapy (400 mg/kg once a week for two weeks and after 14 and 21 days reduced at 200 mg/kg). In addition, linezolid and daptomicin were administered for MRSA positive blood culture. A594V in UL97 gene was re-confirmed in blood sample. At week 30, HCMV viral load increased to 16,314,793 IU/mL.

At week 32 post-transplant, oral LMV treatment was started in monotherapy at a dose of 480 mg daily because of persisting neutropenia and a previous renal toxicity during FOS treatment. HCMV-specific T-cell response was still undetectable at that time when in presence of 76 CD4 and 67 CD8 absolute T cells counts/µl.

At LMV baseline, HCMV DNAemia was 2,800,980 IU/mL. After two weeks of therapy, HCMV DNAemia decreased to 461,160 IU/mL. Five weeks after starting LMV HCMV DNAemia was 42,336 IU/mL. Unfortunately, after 7 and 8 weeks of treatment HCMV DNA increased to 244,566 IU/mL and 703,080 IU/mL, respectively (Fig. 1). Thus, HCMV UL51, UL56 and UL89 genes encoding the terminase complex verify the potential emergence of LMV-resistance-associated mutation [12]. Mutation C325Y in HCMV UL56 terminase gene conferring high-level resistance to LMV [8, 13] was reported. Retrospectively, it was revealed that the mutation was already present as mixture with the wild type after 5 weeks LMV treatment. The patient was treated with CDV at the dosage of 5 mg/kg once a week. After 1-week CDV treatment, HCMV DNA increased up to 2,563,208 IU/mL. Emergence of CDV resistance was excluded by UL54 gene sequence analysis. The patient died after two weeks due to complications caused by HCMV infection.

## Discussion and conclusions

HCMV infection and the associated tissue diseases are still important causes of morbidity and mortality in immunocompromised-transplanted patients, especially in subjects at high risk such as HCMV-seronegative recipients who receive an organ from an HCMV-seropositive donor (D+/R−), due to the lack of prior HCMV immune response [14]. Limited treatment options are available when side effects like nephrotoxicity, electrolyte disturbances, and myelotoxicity or drug resistance occur. Recent studies indicated that LMV might be an important addition to the current strategies in HCMV disease as salvage therapies in SOTRs [15, 16]. LMV appears to be a good alternative to other antiviral especially in view of the favorable safety [17]. However, mutations conferring resistance to LMV have been reported both in HSCT and in SOT recipients [17–20] as soon as after 6 weeks of treatment [18]. Similarly, Hofmann and colleagues reported C351Y mutation in two patients treated with LMV after emergence of GCV-resistance. In both cases, a mismatch D/R was reported [21].

In the case here described, an even faster breakthrough of resistant HCMV during third-line treatment with LMV is reported. In this patient, congenital immunodeficiency associated with iatrogenic immunosuppression, primary HCMV infection likely concurred to the emergence of the GCV-resistance viral strain. Furthermore, LMV could only be administered in monotherapy due to significant myelo- and renal toxicity. It might be suggested that administration of LMV in higher dosage could be more effective, however in a recent study selection of LMV resistance was reported in two patients treated with 720 mg and 960 mg respectively [22]. Therefore, the use of LMV alone might be not sufficient to impair high level of HCMV replication [17, 18, 21]. Moreover, according to international consensus guidelines, monitoring of cell-mediated response should be included in management of solid organ transplant recipients [14].

In conclusion, a warning on the use of LMV monotherapy as rescue treatment for HCMV GCV-resistant infections in transplant recipients is warranted; thus combination therapy, if the patient’s condition permits, may provide better results in case of high viral load.

## Data Availability

All data generated or analysed during this study are included in this published article.

## Abbreviations

GCV: Ganciclovir
vGCV: Valganciclovir
FOS: Foscarnet
LMV: Letermovir
HCMV: Human cytomegalovirus
CVID: Common variable immunodeficiency
